# The impact of a high-fat diet in mice is dependent on duration and age, and differs between muscles

**DOI:** 10.1242/jeb.217117

**Published:** 2020-03-20

**Authors:** Guy A. M. Messa, Mathew Piasecki, Josh Hurst, Cameron Hill, Jason Tallis, Hans Degens

**Affiliations:** 1 Department of Life Sciences, Research Centre for Musculoskeletal Science & Sports Medicine, Manchester Metropolitan University, Manchester M1 5GD, UK; 2 Clinical, Metabolic and Molecular Physiology, MRC-ARUK Centre for Musculoskeletal Ageing Research and National Institute for Health Research (NIHR) Nottingham Biomedical Research Centre, University of Nottingham, Nottingham NG7 2UH, UK; 3 Center for Sport, Exercise and Life Sciences, Alison Gingell Building, Coventry University, Priory Street, Coventry CV1 5FB, UK; 4 Randall Centre for Cell and Molecular Biophysics, New Hunt's House, Guy's Campus, Kings College, London SE1 1UL, UK; 5 Institute of Sport Science and Innovations, Lithuanian Sports University, LT-44221 Kaunas, Lithuania; 6 University of Medicine and Pharmacy of Targu Mures, Târgu Mureş 540139, Romania

**Keywords:** Capillarization, Capillary domains, Diaphragm, Intramyocellular lipid, Soleus

## Abstract

Prolonged high-fat diets (HFDs) can cause intramyocellular lipid (IMCL) accumulation that may negatively affect muscle function. We investigated the duration of a HFD required to instigate these changes, and whether the effects are muscle specific and aggravated in older age. Muscle morphology was determined in the soleus, extensor digitorum longus (EDL) and diaphragm muscles of female CD-1 mice from 5 groups: young fed a HFD for 8 weeks (YS-HFD, *n*=16), young fed a HFD for 16 weeks (YL-HFD, *n*=28) and young control (Y-Con, *n*=28). The young animals were 20 weeks old at the end of the experiment. Old (70 weeks) female CD-1 mice received either a normal diet (O-Con, *n*=30) or a HFD for 9 weeks (OS-HFD, *n*=30). Body mass, body mass index and intramyocellular lipid (IMCL) content increased in OS-HFD (*P*≤0.003). In the young mice, this increase was seen in YL-HFD and not YS-HFD (*P*≤0.006). The soleus and diaphragm fibre cross-sectional area (FCSA) in YL-HFD was larger than that in Y-Con (*P*≤0.004) while OS-HFD had a larger soleus FCSA compared with that of O-Con after only 9 weeks on a HFD (*P*<0.001). The FCSA of the EDL muscle did not differ significantly between groups. The oxidative capacity of fibres increased in young mice only, irrespective of HFD duration (*P*<0.001). High-fat diet-induced morphological changes occurred earlier in the old animals than in the young, and adaptations to HFD were muscle specific, with the EDL being least responsive.

## Abstract (precis)

**Summary:** High-fat diet induces muscle fibre hypertrophy, an increase in oxidative capacity and accumulation of intracellular lipids, earlier in old than in young mice; these adaptations are muscle specific with the EDL being least responsive.

## INTRODUCTION

More than 1.9 billion adults (approximately 24.6% of the world's population) over the age of 18 are overweight or obese (WHO, 2018). Under obesogenic conditions, such as excessive and prolonged consumption of high-fat diets (HFDs), energy intake exceeds expenditure, causing an accumulation of lipids in adipose tissues and ectopically in non-adipose tissues, including skeletal muscle where excess lipids are stored as intramyocellular lipids (IMCLs; lipids within the muscle fibre) (Addison et al., 2014; Unger et al., 2010). In addition to the negative effects of systemic inflammation and insulin resistance associated with obesity (Cesari et al., 2005) on myogenesis and muscle function, muscular lipid accumulation itself can also compromise excitation–contraction coupling, metabolism and contractile function of skeletal muscle (Bonen et al., 2015; Choi et al., 2016; Hancock et al., 2008; Montgomery et al., 2017; Tallis et al., 2018).

The prevalence of sarcopenic obesity, the presence of sarcopenia and obesity in an individual, is increasing in the Western world (Batsis and Villareal, 2018). While the absolute strength and mass of postural and locomotor muscles may be larger in obese than in non-obese individuals (Abdelmoula et al., 2012; Maffiuletti et al., 2007; Tomlinson et al., 2014), probably due to the higher load on the postural muscles during standing and locomotion (Garcia-Vicencio et al., 2016), muscle strength normalized to body mass is lower in obese than in non-obese adolescents (Lee et al., 2012), and young (Maffiuletti et al., 2007) and old adults (Tomlinson et al., 2014; Zoico et al., 2004). In addition, the specific tension (force per cross-sectional area) may be lower in obese individuals, as seen in *in vitro* rodent studies (Hurst et al., 2019; Tallis et al., 2017b) and in human studies (Erskine et al., 2017). Furthermore, it has been reported that the age-related loss of muscle strength is greater in obese than in non-obese women (Tomlinson et al., 2014).

The effects of obesity on skeletal muscle are not systemic, as reflected by the different response in locomotory and respiratory muscles (Tallis et al., 2018). In a study on obese men and women, the predominant upper-body fat distribution was not associated with a significant impairment of respiratory muscle strength (Magnani and Cataneo, 2007). In the lower leg, however, muscle strength is at least partly attributable to a lower specific tension (maximal muscle force per cross-sectional area) in obese older adults (Choi et al., 2016) and obese adult rodents (Kemmochi et al., 2018; Tallis et al., 2018). Part of the lower specific tension may be caused by a larger volume fraction of IMCL, that may be as high as 5% of the muscle fibre volume in obese individuals (Malenfant et al., 2001).

In addition to a decrease in force-generating capacity, fatigue resistance has also been reported to be reduced in obese people (Maffiuletti et al., 2007). Given the positive relationship between muscle fatigue resistance and oxidative capacity in motor units and single muscle fibres (Degens and Veerkamp, 1994), part of the lower fatigue resistance in skeletal muscle of obese people may be due to a reduced oxidative capacity (Crunkhorn et al., 2007; Koves et al., 2005; Shortreed et al., 2009a; Sparks et al., 2005). However, several studies in humans and rodents showed an increase in oxidative capacity with HFD (Garcia-Roves et al., 2006; Hancock et al., 2008; Iossa et al., 2002; Li et al., 2016a; Miller et al., 1984; Sadler et al., 2012), rather than a decrease. This discrepancy may be due to differences in diet duration as the effects of a HFD are time dependent: a 4 week period of HFD resulted in little effect on the extensor digitorum longus (EDL) oxidative capacity, but a significant increase after a 12 week period (Eshima et al., 2017). In addition, adaptations to a HFD may be muscle specific and dependent on age. For instance, in young-adult mice, it has been reported that fatigue resistance is reduced in the EDL, but not in the diaphragm and soleus muscle (Hurst et al., 2019), while in old mice, 9 weeks of HFD did not induce any changes in fatigue resistance in the EDL, soleus or diaphragm.

A HFD-induced shift from glucose to fatty acid metabolism may necessitate an enhanced oxygen supply as fatty acid oxidation requires approximately 8% more oxygen than carbohydrate oxidation for each ATP molecule generated (Silvennoinen et al., 2013). In mice, this shift in metabolism has been suggested to stimulate angiogenesis as reflected by the higher capillary density (CD) and capillary to fibre ratio (C:F) in gastrocnemius and quadriceps femoris muscles after 19 weeks HFD (Silvennoinen et al., 2013), which may be stimulated via the leptin-induced increase in VEGF-A expression (Nwadozi et al., 2019). Others, however, found no difference in the C:F or CD in the plantaris muscle of rats fed a HFD for 8 weeks (Roudier et al., 2009), and it may thus be that only after prolonged exposure to a HFD does angiogenesis occur. The effects of a HFD on the heterogeneity of capillary spacing, which has an important impact on tissue oxygenation (Degens et al., 2006), is yet to be elucidated.

The majority of current available data on the effects of a HFD on skeletal muscle morphology do not consider aged animals (Bonnard et al., 2008; de Wilde et al., 2008; Eshima et al., 2017; Nwadozi et al., 2016; Silvennoinen et al., 2013). However, one study that examined the effect of an extended HFD on *in vivo* skeletal muscle morphology and lipid content in aged rats reported an inverse association between hindlimb muscle volume and muscular lipid content (Bollheimer et al., 2012). This observation suggests that high-fat feeding and subsequent elevated muscular lipids may contribute to age-related muscle atrophy.

The aim of the present study was to comprehensively analyse the effects of a HFD and the duration of HFD on the morphology of the soleus, EDL and diaphragm muscles in mature (20 weeks old) and early ageing (79 weeks old) mice. We hypothesized that a HFD (1) induces in all muscles an increase in IMCL, and (2) that the locomotory muscles will show a decrease in oxidative capacity and capillarization, while (3) the diaphragm will show an increase in oxidative capacity and capillarization, where (4) the HFD-induced changes will increase with duration of feeding and (5) be more pronounced at old age.

## MATERIALS AND METHODS

### Animals and diets

We compared the effect of HFD in the diaphragm, soleus and EDL in female CD-1 mice (Charles River, Harlan Laboratories, UK) that were 20 or 79 weeks old at the end of the experiment. The 79 week old female mice are a model of early ageing, where survival is 50% (Navarro et al., 2002) and the muscles show morphological changes suggestive of early ageing (Messa et al., 2019), further supported by a significant decline in soleus and EDL specific tension and specific power compared with 10 week old animals (Hill et al., 2018). In addition, the CD-1 stock is heterogeneous and therefore has greater genetic variability than many other strains of lab mice (Aldinger et al., 2009; Rice and O'Brien, 1980) and as such reflects the genetic variability seen in humans.

The female CD-1 mice were housed and aged at Coventry University in cages of 8–10 individuals on a 12 h:12 h light:dark cycle at 50% relative humidity. Four week old (*n*=56) and 68 week old (*n*=60) mice were randomly allocated to either HFD or normal diet (see below).

The 4-week-old mice (young, Y) were provided with normal chow (Y-Con, *n*=28) or a HFD for 16 weeks (YL-HFD, *n*=28); an additional group of mice was provided with a HFD for 8 weeks from the age of 12 weeks (YS-HFD, *n*=16).

Animals used for the ageing element of the study (old, O) were purchased at 9 weeks of age and provided with a standard lab chow only during ageing. At the age of 70 weeks they either continued with regular chow (O-Con, *n*=30) or were given a HFD (OS-HFD, *n*=30) for a duration of 9 weeks.

All experimental groups fed a HFD were simultaneously provided with the standard lab chow in the form of a self-selected forage diet (Hill et al., 2019; Hurst et al., 2019). All groups had *ad libitum* access to each diet and water.

The caloric composition of the standard chow was: protein 17.5%, fat 7.4%, carbohydrate 75.1%; gross energy 3.52 kcal g^−1^; metabolizable energy 2.57 kcal g^−1^ [CRM(P) SDS/Dietex International Ltd, Whitham, UK]. The caloric composition of the HFD was: protein 18.0%, fat 63.7%, carbohydrate 18.4%; gross energy 5.2 kcal g^−1^; metabolizable energy 3.8 kcal g^−1^ (Advanced Protocol PicoLab, Fort Worth, TX, USA).

At the age of 20 or 79 weeks, mice were killed by cervical dislocation. All experimental procedures were carried out in compliance with the local ethical review of Coventry University under a UK Home Office project licence held in accordance with the Animals (Scientific Procedures) Act 1986. Animals were weighed, and snout-to-anus length determined to calculate the body mass index (BMI), as body mass (kg) divided by length (cm) squared.

The removal and processing for analysis of muscle morphology of the soleus, EDL and right part of the diaphragm muscles were performed as outlined previously (Messa et al., 2019; Tallis et al., 2017b). After excision, the muscles were blotted dry, weighed, embedded in Tissue-Tek freezing medium (Leica Biosystems, Nußloch, Germany), frozen in liquid nitrogen-cooled isopentane (Sigma-Aldrich, Steinheim, Germany) and stored at −80°C. Only one muscle per animal was collected.

### Histological analysis and microscopy

Serial 10 µm thick cross-sections of the soleus, EDL and diaphragm muscles were cut with a cryostat (CM3050S; Leica) at −21°C and collected on Superfrost Plus microscope slides. Serial sections were stained for IMCL, myosin heavy chain (MHC), capillaries or succinate dehydrogenase (SDH) (Messa et al., 2019).

#### IMCL

Sudan Black B was utilized to stain IMCL; this dye stains mainly neutral lipids (primarily triglycerides) with a blue-black tint. Briefly, air-dried sections were fixed in 10% formalin for 10 min. Sections were then washed 3 times for 1 min in distilled water before incubation in propylene glycol for 3 min. Sections were then incubated in Sudan Black B solution (preheated at 60°C) for 7 min, differentiated in 85% propylene glycol for 3 min and subsequently washed 3 times for 1 min in distilled water. Sections were cover-slipped using glycerol gelatin.

#### Fibre typing

Serial sections were immunohistochemically stained for type I, IIa, IIx or IIb MHC using mouse monoclonal primary antibodies BA-D5 (supernatant; 1 µg ml^−1^), SC-71 (supernatant; 1 µg ml^−1^), 6H1 (supernatant; 10 µg ml^−1^) and BF-F3 (supernatant; 5 µg ml^−1^), respectively (Developmental Studies Hybridoma Bank, Iowa City, IA, USA). One section was co-stained for type I, IIa and IIx MHC and a serial section for type IIb MHC.

Sections were fixed with ice-cold acetone for 15 min and then blocked for 45 min with 10% goat serum in phosphate-buffered saline (PBS) at room temperature. Following washing with PBS, the sections were incubated with the primary antibody for 90 min in a humid chamber. The sections were subsequently washed in PBS and incubated in the dark for 60 min with Alexa Fluor 350 goat anti-mouse IgG2_b_ (A-21140; 2 µg ml^−1^, Invitrogen) and Alexa Fluor 488 goat anti-mouse IgG_1_ (A-21121; 2 µg ml^−1^, Invitrogen) for type I and IIa fibres, respectively, and Alexa Fluor 555 goat anti-mouse IgM (A-21426; 2 µg ml^−1^, Invitrogen) for type IIx and IIb fibres. Sections were washed, dried and mounted using Prolong Diamond anti-fade mounting medium (Life Technologies). Sections without the primary antibodies served as negative controls. Images were taken with a Carl Zeiss Axio MRc Camera (Göttingen, Germany) on a Zeiss fluorescence microscope (10× objective).

#### Capillary staining

Capillaries were visualized using lectin as described previously (Ballak et al., 2016). Briefly, air-dried sections were fixed with ice-cold acetone for 15 min, and blocked with 0.1% bovine serum albumin (BSA) diluted in Hepes for 60 min. Subsequently, the sections were treated with a peroxide solution for 30 min and incubated with biotinylated *Griffonia* (*Bandeira*) *simplicifolia* lectin (GSL I; Vector Laboratories, Peterborough, UK; 50 µg ml^−1^ diluted in 1% BSA/Hepes) for 60 min. A 5 min wash was conducted between each step. Sections were then treated with avidin-biotinylated horseradish peroxidase (Vectastain ABC kit, Vector Laboratories) for 60 min, washed with Hepes, and incubated with horseradish peroxidase substrate diaminobenzidine (Vectastain DAB kit, Vector Laboratories) for 5 min. After a wash in distilled water, the sections were mounted in glycerol gelatin (Sigma-Aldrich, Aldrich, UK).

#### SDH

**Fig. 1.**
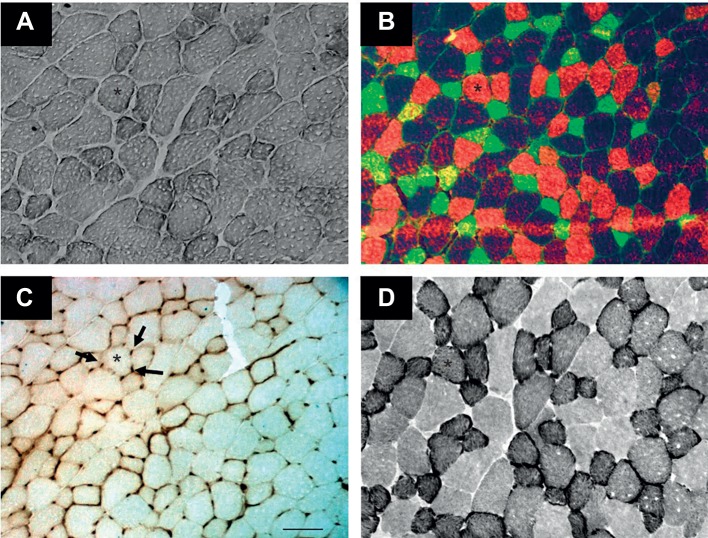
**Serial sections of extensor** **digitorum longus (EDL) from a 79** **week old mouse fed a high-fat diet (HFD).** Sections were stained for (A) intramyocellular lipid (IMCL), (B) myosin heavy chain (MHC), (C) capillaries (arrows) and (D) succinate dehydrogenase (SDH) activity. The asterisk indicates the same fibre in the four panels. Green, type IIa; red, type IIx; and unstained, type IIb fibres. Scale bar: 100 µm.

SDH activity was assessed according to the protocol described by Wüst et al. (2009). Sections were incubated in 37.5 mmol l^−1^ sodium phosphate buffer (pH 7.6), 74 mmol l^−1^ sodium succinate and 0.4 mmol l^−1^ Tetranitro Blue Tetrazolium in the dark at 37°C for 20 min. The reaction was stopped with 0.01 mol l^−1^ hydrochloric acid for 10 s. Then, the slides were washed with two changes of distilled water, mounted in glycerol–gelatin and stored in the dark until measurement of the staining intensity within 2 days. All samples were processed simultaneously in the same incubation solution, ensuring that all samples were subjected to the same conditions. Characteristic staining of EDL in an old mouse is shown in Fig. 1.

### Morphometry analysis

Stained sections were photographed with a digital camera (Zeiss AxioCam MRc) on a light microscope (Carl Zeiss, Göttingen, Germany; 20× objective). At least two images per muscle cross-section were taken and 245±74 complete fibres were analysed per sample.

#### IMCL

The IMCL content of individual fibres was determined using a microscope with a 20× objective and bright-field settings. Images were digitally captured using a black and white AxioCam ICMI camera (Göttingen, Germany) and analysed with ImageJ (National Institutes of Health, USA, https://imagej.nih.gov/ij/). The fibre of interest was outlined, and the grey levels were converted to optical density (OD) using a calibration curve constructed from a series of filters of known OD. For each section, a separate calibration curve was constructed, and all images were taken at the same exposure with the same microscope settings. The OD of the Sudan Black B stain was determined in individual fibres and the background OD for each fibre was subtracted from the OD measured. The higher the net OD for the Sudan Black B stain, the higher the IMCL in the fibre.

#### Fibre-type composition and fibre size

The fibre outlines and capillary centres were collected with a digitizing program (BTablet, BaLoH Software, Ooij, The Netherlands, http://www.baloh.nl) and the data analysed with AnaTis (BaLoH Software). The fibre cross-sectional area (FCSA) was calculated for each fibre. The fibre-type composition was expressed as number percentage and as area percentage (area occupied by an individual fibre type divided by the total area occupied by all fibres).

#### Capillarization

The capillarization in the muscles was determined with the method of capillary domains (Degens et al., 2006; Hoofd et al., 1985) using AnaTis. Briefly, a capillary domain is defined as the area of a muscle cross-section surrounding an individual capillary delineated by equidistant boundaries from adjacent capillaries. The capillary domain provides a good estimate of the capillary oxygen supply area, even in muscles with mixed fibre-type composition (Al-Shammari et al., 2014). In addition to the overall parameters of muscle capillarization, including capillary density (CD; number of capillaries per mm^2^) and the capillary to fibre ratio (C:F), this method enables the identification of the capillary supply to individual fibres even when they lack direct capillary contact. The local capillary to fibre ratio (LCFR), the sum of the fractions of the capillary domains overlapping a particular fibre, provides a continuous rather than a discrete value of the capillary supply to a fibre and takes into consideration that a capillary supplies more than one fibre (Barnouin et al., 2017). The ratio of LCFR to FCSA provides the capillary density for a given fibre, defined as the capillary fibre density (CFD). The radius (*R*) of a domain, calculated from a circle with the same surface area, provides an indication of the maximal diffusion distance from the capillary to the edge of its domain. *R* shows a lognormal distribution, and thus the log*_R_*SD (logarithmic standard deviation of the domain radius) is a measure of the heterogeneity of capillary spacing, which plays a vital role in muscle oxygenation (Degens et al., 2006; Hoofd et al., 1985).

#### SDH

Photomicrographs of stained sections of SDH were taken with a light microscope with a 660 nm interference filter and a white and black AxioCam ICM1 camera. All images were taken at the same exposure with the same microscope settings. Images were analysed using ImageJ (National Institutes of Health, USA). To measure the OD of a given fibre, the outline of the fibre was drawn and the background OD subtracted. For each session, a separate calibration curve was made with filters of known OD (*A*_660_). The calibration curve was used to convert the absorbance values of the SDH staining into OD values.

To assess the SDH activity (SDH-OD), the OD (*A*_660_) was converted to the rate of staining and expressed as the increase in absorbance at 660 nm (*A*_660_) per micrometre section thickness per second of incubation time (Δ*A*_660_ µm^−1^ s^−1^). The SDH-OD multiplied by the FCSA yielded the integrated SDH activity (SDH-Int in Δ*A*_660_ μm s^−1^):(1)
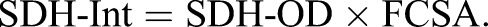
It has been shown that the mass-specific maximal oxygen uptake (*V̇*_O_2_,max_ in ml kg^−1^ min^−1^) is proportional to SDH-OD and that SDH-Int is linearly related to the maximum rate of oxygen uptake (*V̇*_O_2_,max,fibre_ in ml min^−1^) of the muscle fibre (van der Laarse et al., 1989).

### Statistical analysis

All statistical analyses were performed using IBM SPSS version 25 (IBM SPSS Statistics for Windows, IBM Corp., Armonk, NY, USA). The Shapiro–Wilk test indicated that all data were normally distributed. A four-way ANOVA with the factors diet, age, muscle and fibre type – where appropriate – was applied and three- and four-way interactions were excluded. If a main effect of diet or interactions with diet was found, LSD *post hoc* tests were performed to locate the differences. To assess to what extent the capillary supply to a fibre was determined by the oxidative capacity of the fibre (SDH-OD), FCSA, fibre type, muscle of origin, age and/or diet, we performed a stepwise regression. Hybrid fibres (type IIax and IIxb) were excluded from the analysis as they occurred infrequently. Statistical significance was accepted at *P*<0.05. Data are expressed as means±s.d.

## RESULTS

Data for control muscles of young-adult and old mice, and the effects of ageing have been published previously (Messa et al., 2019), and the ageing comparison is not repeated here. However, we did consider age×diet interactions, where a significant interaction indicates that the response to a HFD differs between young-adult and old mice.

In many cases, the collected muscles were not suitable for histological analysis because of damage incurred during preparation, freezing artefacts and/or large areas where muscle fibres were cut longitudinally – conditions that precluded adequate morphological analysis. Because of this, no data were collected for the EDL from YS-HFD mice. Nevertheless, we included them in Table 1, as they were still applicable when calculating body and muscle mass.

**Table 1.**
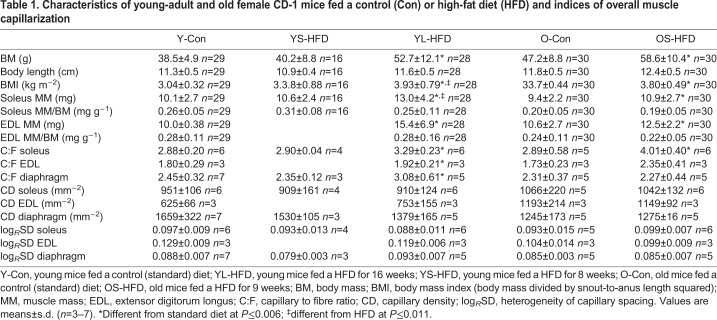
**Characteristics of young-adult and old female CD-1 mice fed a control (Con) or high-fat diet (HFD) and indices of overall muscle capillarization**

### Mouse characteristics

In young-adult mice, an increase in body mass, BMI and soleus and EDL mass was only seen after 16 weeks of HFD (YL-HFD group; *P*≤0.001; Table 1), with no significant differences from control (Y-Con) after 8 weeks of HFD (YS-HFD group). In old mice, 9 weeks of a HFD induced an increase in body mass, BMI and soleus and EDL mass (*P*≤0.001; Table 1), while the muscle mass to body mass ratio was not significantly different between the OS-HFD and O-Con groups. There were no significant diet×fibre type interactions for any of the parameters, indicating that fibres of all types responded similarly to diet. Body length did not differ significantly between the HFD and control mice.

### IMCL levels

**Fig. 2.**
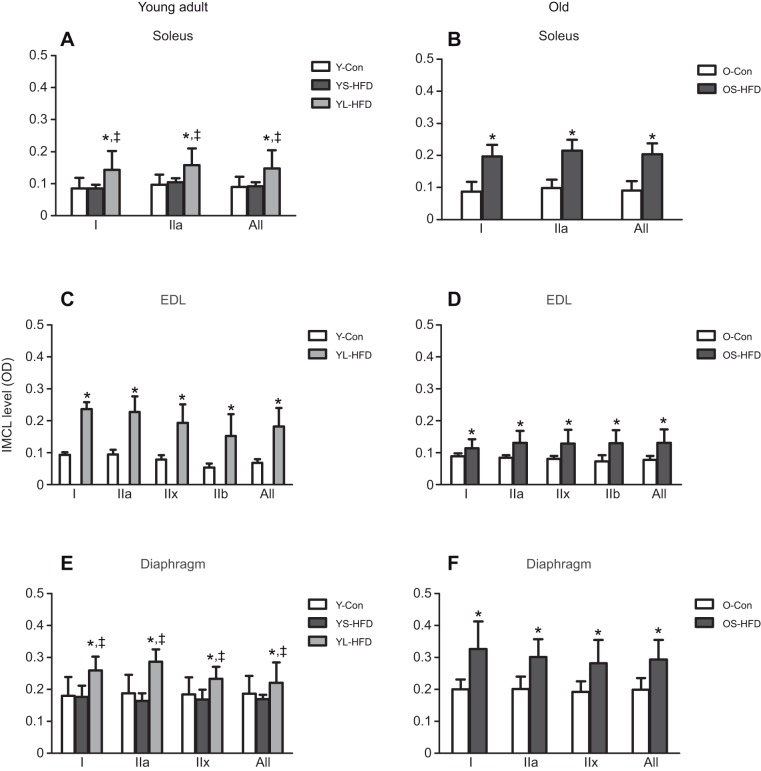
**Intramyocellular lipids** **(IMCLs) in muscles of control mice and those fed a HFD.** (A,B) Soleus, (C,D) EDL and (E,F) diaphragm muscles of young (20 weeks old; left) and old (79 weeks old; right) control mice (Y-Con and O-Con, respectively) and mice fed a HFD for 8–9 weeks (YS-HFD and OS-HFD, respectively) or 16 weeks (young mice only, YL-HFD). *Different from control; ^‡^different from YS-HFD at *P*≤0.006. Values are means±s.d. (*n*=3–7). OD, optical density.

In young-adult mice, IMCL levels were elevated above Y-Con after 16 weeks of HFD (YL-HFD), but not after 8 weeks of HFD (YS-HFD), in the soleus, EDL and diaphragm (*P*≤0.006). In the old mice, 9 weeks of HFD resulted in an elevated IMCL in all three muscles (*P*≤0.003; Fig. 2).

### Muscle fibre-type composition and FCSA

#### Fibre-type composition

**Fig. 3.**
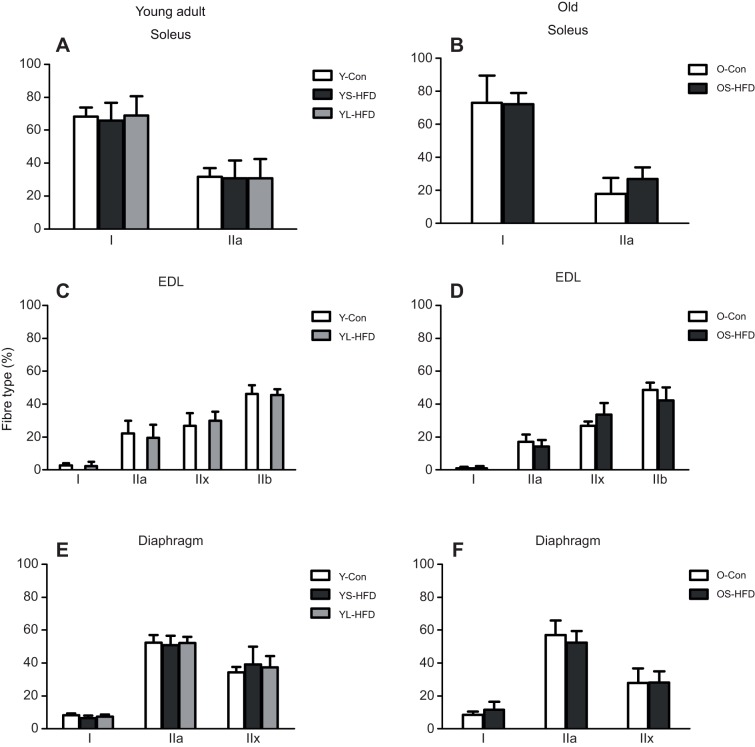
**Fibre-type composition in muscles of control mice and those fed a HFD.** (A,B) Soleus, (C,D) EDL and (E,F) diaphragm muscles of young (20 weeks old; left) and old (79 weeks old; right) control mice (Y-Con and O-Con, respectively) and mice fed a HFD for 8–9 weeks (YS-HFD and OS-HFD, respectively) or 16 weeks (young mice only, YL-HFD). Values are means±s.d. (*n*=3–7).

Fig. 3 shows that the fibre-type composition in the soleus (Fig. 3A,B), EDL (Fig. 3C,D) and diaphragm (Fig. 3E,F) muscles was not significantly affected by either 8–9 or 16 weeks of HFD in either young-adult or old mice.

#### FCSA

**Fig. 4.**
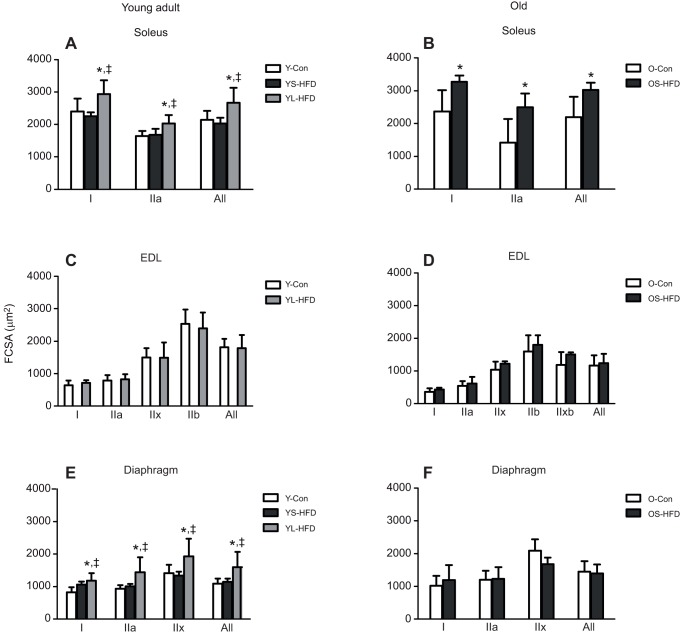
**Fibre cross-sectional area (FCSA) in muscles of control mice and those fed a HFD.** (A,B) Soleus, (C,D) EDL and (E,F) diaphragm muscles of young (20 weeks old; left) and old (79 weeks old; right) control mice (Y-Con and O-Con, respectively) and mice fed a HFD for 8–9 weeks (YS-HFD and OS-HFD, respectively) or 16 weeks (young mice only, YL-HFD). *Different from control; ^‡^different from YS-HFD at *P*≤0.004. Values are means±s.d. (*n*=3−7).

Young-adult animals fed a HFD for 16 weeks had larger FCSA in the soleus and diaphragm (*P*≤0.004); however, no significant difference in FCSA was seen between Y-Con mice and mice fed a HFD for 8 weeks (YS-HFD; Fig. 4A,E). Old mice had a larger FCSA in the soleus after 9 weeks on a HFD (OS-HFD; *P*<0.001; Fig. 4B). In the EDL muscle, no significant differences in FCSA were observed between HFD and control groups in either young-adult or old mice (Fig. 4C,D).

### SDH activity

**Fig. 5.**
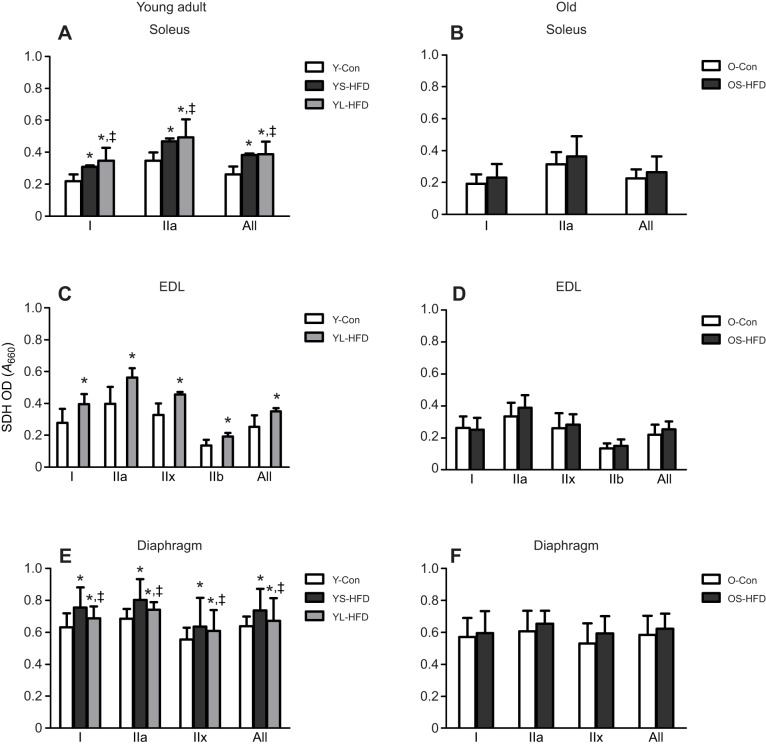
**Succinate dehydrogenase activity (SDH-OD) in muscles of control mice and those fed a HFD.** (A,B) Soleus, (C,D) EDL and (E,F) diaphragm muscles of young (20 weeks old; left) and old (79 weeks old; right) control mice (Y-Con and O-Con, respectively) and mice fed a HFD for 8–9 weeks (YS-HFD and OS-HFD, respectively) or 16 weeks (young mice only, YL-HFD). *Different from control at *P*<0.001; ^‡^different from YS-HFD at *P*≤0.025. Values are means±s.d. (*n*=3–7).

**Fig. 6.**
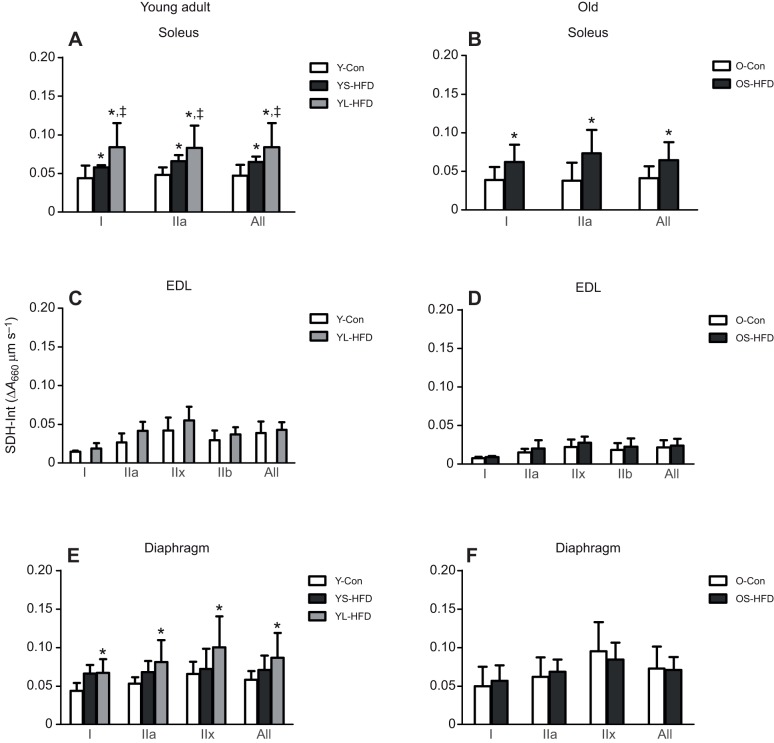
**Integrated SDH activity (SDH-Int) in muscles of control mice and those fed a HFD.** (A,B) Soleus, (C,D) EDL and (E,F) diaphragm muscles of young (20 weeks old; left) and old (79 weeks old; right) control mice (Y-Con and O-Con, respectively) and mice fed a HFD for 8–9 weeks (YS-HFD and OS-HFD, respectively) or 16 weeks (young mice only, YL-HFD). *Different from control at *P*≤0.008; ^‡^different from YS-HFD at *P*≤0.033. Values are means±s.d. (*n*=3–7).

In young-adult mice, mass-specific SDH activity (SDH-OD) was higher in the soleus, EDL and diaphragm muscle from animals in the HFD versus control group, irrespective of duration of HFD (*P*<0.001; Fig. 5A,C,E), but no significant effect of HFD was seen in the old animals (Fig. 5B,D,F). In both young-adult and old mice, SDH-Int in the EDL was not significantly different between animals fed a HFD or control diet (Fig. 6C,D). In young-adult mice, SDH-Int level of fibres in the soleus muscle was YL-HFD>YS-HFD>Y-Con (Fig. 6A; *P*≤0.033) and in the diaphragm YL-HFD>Y-Con (Fig. 6E; *P*<0.001). In old mice, SDH-Int in the soleus was also higher in OS-HFD than in OS-Con (Fig. 6B; *P*=0.008), but no significant effect of HFD was seen in the diaphragm of the old animals (Fig. 6F).

### Muscle capillarization

#### Indices of global capillary supply

In the young animals, 16 weeks but not 8 weeks of HFD induced a significant increase in the C:F ratio in all three muscles (*P*≤0.006; Table 1). In the old animals, 9 weeks of HFD induced an increase in C:F ratio in the soleus muscle only (*P*=0.006; Table 1). There were no significant differences in the CD between control animals and animals fed a HFD for 8–9 or 16 weeks in any of the muscles from young-adult or old animals (Table 1). There was also no significant difference in the log*_R_*SD between control and HFD in any of the muscles (Table 1).

#### LCFR

**Fig. 7.**
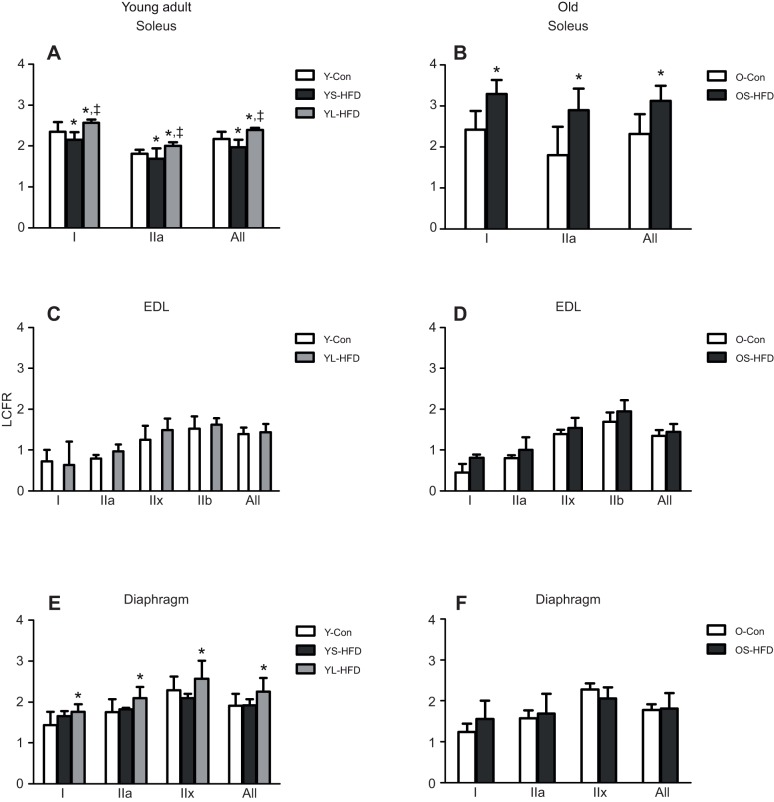
**Local capillary to fibre ratio (LCFR) in muscles of control mice and those fed a HFD.** (A,B) Soleus, (C,D) EDL and (E,F) diaphragm muscles of young (20 weeks old; left) and old (79 weeks old; right) control mice (Y-Con and O-Con, respectively) and mice fed a HFD for 8–9 weeks (YS-HFD and OS-HFD, respectively) or 16 weeks (young mice only, YL-HFD). *Different from control at *P*≤0.016; ^‡^different from YS-HFD at *P*=0.045. Values are means±s.d. (*n*=3–7).

In young animals, there was a main effect of diet on LCFR (*P*<0.001; Fig. 7). However, the muscle×diet interaction (*P*=0.003) indicated that the effect of diet differed between muscles. In the soleus muscle of young mice, the LCFR in animals fed a HFD for 8 weeks (YS-HFD) was lower than that in Y-Con (*P*=0.045) and was higher in mice fed a HFD for 16 weeks than in both Y-Con and YS-HFD mice (Fig. 7A; *P*≤0.005). In the soleus of old mice, 9 weeks on a HFD resulted in a higher LCFR (Fig. 7B; *P*<0.001). *Post hoc* analysis revealed that in the EDL of both young and old animals, the LCFR did not differ significantly between animals on a control or HFD (Fig. 7C,D). The LCFR of fibres in the diaphragm was higher in mice fed a HFD for 16 weeks (YL-HDF) than in Y-Con (*P*=0.016). There was no significant difference in diaphragm LCFR between control and 8–9 weeks HFD in either young (YS-HFD) or old (OS-HFD) animals (Fig. 7E,F).

#### CFD

**Fig. 8.**
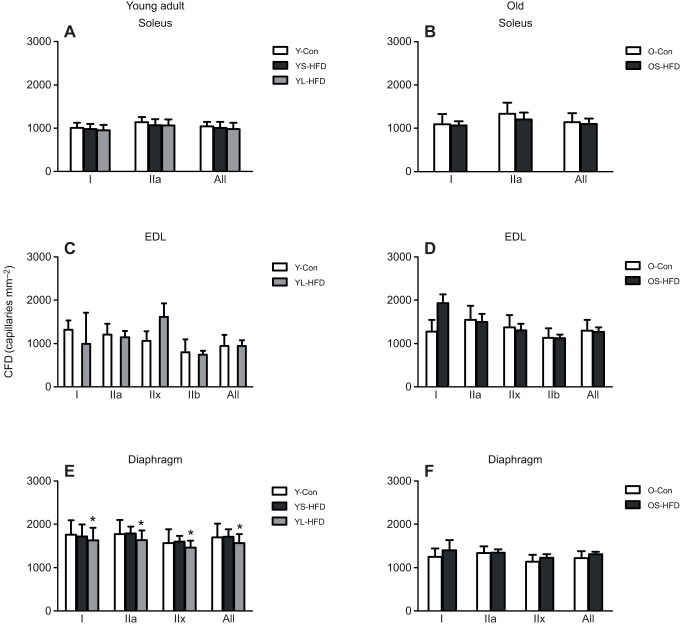
**Capillary fibre density (CFD) in muscles of control mice and those fed a HFD.** (A,B) Soleus, (C,D) EDL and (E,F) diaphragm muscles of young (20 weeks old; left) and old (79 weeks old; right) control mice (Y-Con and O-Con, respectively) and mice fed a HFD for 8–9 weeks (YS-HFD and OS-HFD, respectively) or 16 weeks (young mice only, YL-HFD). *Different from control at *P*=0.003. Values are means±s.d. (*n*=3–7).

There was no main effect of diet on CFD in either young or old animals (Fig. 8). However, the diet×muscle interaction in young animals (*P*=0.008) was explained by a higher CFD in the diaphragm of young animals fed a HFD for 16 weeks (YL-HFD) than in control mice or those fed a HFD for 8 weeks (YS-HFD).

### Determinants of fibre capillary supply

**Fig. 9.**
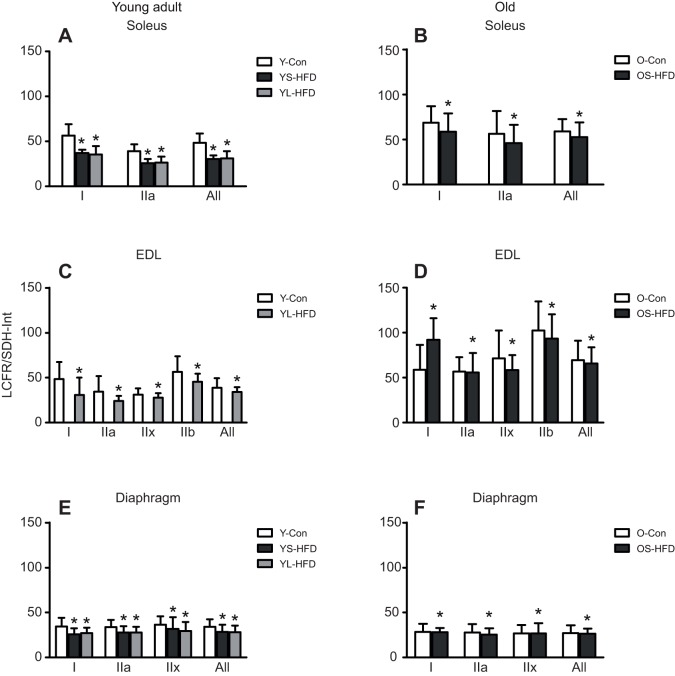
**LCFR:SDH-Int ratio in muscles of control** **mice and those fed a HFD.** (A,B) Soleus, (C,D) EDL and (E,F) diaphragm muscles of young (20 weeks old; left) and old (79 weeks old; right) control mice (Y-Con and O-Con, respectively) and mice fed a HFD for 8–9 weeks (YS-HFD and OS-HFD, respectively) or 16 weeks (young mice only, YL-HFD). *Main effect of diet *P*=0.003. Values are means±s.d. (*n*=3–7).

To assess differences between muscles, fibre types and diet in the matching of oxygen supply (LCFR) and demand (SDH-Int) of a fibre, LCFR/SDH-Int was calculated as a measure of the supply:demand ratio. A HFD led to a decrease in LCFR/SDH-Int of muscle fibres (*P*=0.003), irrespective of duration of HFD, age, fibre type and muscle of origin (Fig. 9), as reflected by the absence of significant interactions of diet with age, muscle and type.

In the soleus, EDL and diaphragm, the LCFR correlated positively with FCSA (*r*^2^=0.46–0.66; *P*<0.001) and this correlation did not differ significantly between control and HFD mice (data not shown).

The contribution of different factors to the LCFR (capillary supply of a fibre) was assessed by performing a stepwise linear regression. The factors included in the model were FCSA, SDH-OD, muscle (soleus, EDL and diaphragm) and diet. The primary determinant of LCFR was FCSA (adjusted *r*^2^=0.670; *P*<0.001), which explained most (67%) of the variance in LCFR. Muscle (adjusted *r*^2^=0.783; *P*<0.001), SDH-OD (adjusted *r*^2^=0.790; *P*<0.001) and diet (adjusted *r*^2^=0.797; *P*<0.001) explained an additional 12.9% of the variance in LCFR, with only a 0.7% contribution of diet.

## DISCUSSION

In the present study, we evaluated whether morphological changes induced by a HFD differed between the soleus, EDL and diaphragm and whether the adaptations differed between young-adult (20 weeks) and old mice (79 weeks). We observed that although the effect of a HFD did not differ between fibre types, the changes in muscle morphology seemed to be least pronounced in the EDL muscle. While increases in body mass and BMI, accumulation of IMCL, and increases in oxidative capacity, angiogenesis and fibre hypertrophy occurred in the diaphragm and soleus muscle, they occurred sooner in old than in young mice. Angiogenesis was in general proportional to the HFD-induced increase in fibre size, but signs of a developing mismatch between oxygen supply and demand were reflected by the lower ratio of capillary number to maximal oxygen consumption of a fibre (reflected by the LCFR:SDH-Int ratio) in animals on a HFD. Thus, older animals were more susceptible to HFD-induced changes in muscle morphology that may result in a mismatch between oxygen supply and demand to the working muscles.

### Effects of HFD on body mass and IMCL levels

The consumption of a HFD resulted in an increased BMI and muscle mass. In line with previous studies (Andrich et al., 2018; Baek et al., 2018; Eshima et al., 2017; Hegarty et al., 2002; Kaneko et al., 2011; Silvennoinen et al., 2013), this was accompanied by elevated IMCL levels in the soleus, diaphragm and EDL of our mice. This HFD-induced increase in body mass, BMI and IMCL occurred after only 9 weeks in old mice, but was only seen after 16 weeks in the young-adult mice, similar to the absence of changes in IMCL after 4 weeks and elevated IMCL levels after 12 weeks of a HFD seen by others in young-adult mice (Eshima et al., 2017). It should be noted, however, that the BMI of the control old mice was higher than that of the young-adult mice, suggesting greater adiposity. As fatty acids are primarily stored in adipocytes, and only when in excess are stored elsewhere (Koutsari et al., 2012), their larger adiposity may cause the earlier deposition of fatty acids in the muscle of old mice. Whatever the explanation, this suggests that old mice are more susceptible to the effects of a HFD.

### Muscle fibre-type composition and FCSA

Our data showed that a HFD did not induce a change in fibre-type composition in any of the muscles, irrespective of age, as has also been observed by others in rodents (Shortreed et al., 2009b; Turpin et al., 2009). Eshima et al. (2017), however, reported an increased proportion of type IIx fibres concomitant with a decrease of type IIb fibres in the EDL muscle of mice on a 12 week HFD from the age of 8 weeks. The discrepancy between their study and ours may be related to the sex and strain of the mice, as we used female CD-1 mice and Eshima et al. (2017) used male C57BL/6J mice. Indeed, it has been found that 52 weeks of HFD caused a decrease of type I fibres in the soleus of male but not female mice (Denies et al., 2014). Changes in fibre-type composition have regularly been given as an explanation for obesity-induced changes in contractile function (Acevedo et al., 2017; Ciapaite et al., 2015; Kaneko et al., 2011). The absence of significant changes in fibre-type composition in our study corresponds with the observation that in both the young-adult (Hurst et al., 2019; Tallis et al., 2017b) and old mice (Hill et al., 2019) there were no significant changes in the velocity of contraction at which peak power occurred or differences in fatigue resistance.

Corroborating previous observations (Bott et al., 2017; Eshima et al., 2017; Shortreed et al., 2009b; Turpin et al., 2009), we observed an increase in soleus and diaphragm FCSA of young-adult mice on a HFD for 16 weeks but not in those on a HFD for 8 weeks. In old mice, the FCSA was already elevated in the soleus after 9 weeks HFD, indicating that the effects of a HFD on soleus FCSA occurred earlier in old than in young-adult mice, again supporting the notion that muscles of old mice are more susceptible to obesity or a HFD.

The hypertrophy in the postural soleus muscle may be an adaptation to the increased loading resulting from an elevated body mass (Konhilas et al., 2005; Terada et al., 2012). Indeed, in both young-adult and old mice, the soleus muscle mass to body mass ratio did not change significantly after HFD. This may also explain why we did not observe a significant change in the FCSA of fibres in the EDL, which does not play a significant antigravity role. In line with this, it has been found that unloading by hindlimb suspension results in greater atrophy in the soleus than in the EDL (Stevens et al., 2000). Nevertheless, there was no commensurate increase in absolute isometric force in the muscles of mice on a HFD (Hill et al., 2019; Hurst et al., 2019; Tallis et al., 2017b), which may be a consequence of an increased proportion of the cytoplasmic volume occupied by IMCL. Indeed, after 12 weeks, but not 4 weeks, on a HFD, the specific tension of muscles was reduced (Eshima et al., 2017; Hurst et al., 2019; Tallis et al., 2017b) in young-adult muscles, but surprisingly not in old muscles (Hill et al., 2019).

In the diaphragm, the FSCA increased with HFD in the young-adult mice only, but not in the old mice. It should be noted, however, that this effect on the FCSA in the diaphragm of the young-adult mice was only seen after 16 weeks on a HFD, and it is thus possible that such an increase in FCSA would also occur with a longer duration of HFD in the old mice. It is possible that the increased FCSA is compensatory hypertrophy to maintain the maximal force and power-generating capacity of the diaphragm in the face of a decreased force and power-generating capacity per muscle mass previously observed in the same animals after a HFD and with ageing (Hill et al., 2019; Hurst et al., 2019).

In summary, the HFD did not induce changes in fibre-type composition, and any increases in FCSA were muscle specific (showing no change in the EDL), occurred earlier in old than in young-adult mice, and were most likely a secondary adaptation to the increased loading due to an increase in body mass, rather than a direct effect of a HFD.

### Oxidative capacity

Here, we showed that a HFD caused an increase in the oxidative capacity (reflected by the SDH-OD) in muscles from young-adult but not old mice. The increase in skeletal muscle oxidative capacity with HFD has been previously reported in young-adult rodents (Eshima et al., 2017; Garcia-Roves et al., 2007; Hancock et al., 2008; Li et al., 2016b; Miller et al., 1984; Sikder et al., 2018; Thomas et al., 2014; Turner et al., 2007). Interestingly, it has been reported that the expression of enzymes in the citric acid cycle, β-oxidation and respiratory chain is comparable to that with standard diet after 2 weeks of HFD, but similarly elevated after 8 and 16 weeks of a HFD (Sadler et al., 2012). Such HFD-induced changes in the expression of oxidative enzymes correspond to our observation of similarly elevated SDH-OD in young-adult mice on a HFD for 8 and 16 weeks. It is possible that in the young animals this elevated oxidative capacity, even in the fast EDL muscle, and associated capacity for β-oxidation may have prevented the early accumulation of IMCL in the young animals, something not seen in the old animals, where indeed IMCL accumulation occurred after 9 weeks HFD.

The increase in SDH-OD (without an increase in IMCL) after 8 weeks of a HFD was somewhat reduced after 16 weeks in the diaphragm. Such a decrease may be due to lower physical activity levels in response to a HFD (Moretto et al., 2017) that would particularly reduce the activity of the respiratory muscles. It may be that lower physical activity levels in old than in young mice (Ingram, 2000; Tallis et al., 2017a) may also underlie the absence of an increase in oxidative capacity in the muscles from old mice. Another possibility is that the increase in muscle oxidative capacity in young-adult mice is at least partly mediated by activation of peroxisome proliferator-activated receptor α (PPARα), which has been shown to induce an increased expression of genes involved in β-oxidation in neonatal cardiomyocytes (van der Lee et al., 2000). The absence of, or attenuated increase in, oxidative capacity in the old mice may then be related to a reduced expression of PPARα, something observed in the heart of old mice (Bonda et al., 2017). Whatever the explanation, these data indicate that while a HFD induces an increase in muscle oxidative capacity in young-adult mice, this is not the case in old mice.

### Capillarization

As discussed above, we found that a HFD induced increases in muscle oxidative capacity in muscles from young-adult mice. This and the reported increase in β-oxidation (Sadler et al., 2012) may reflect a shift from glucose to fatty acid metabolism. Given that per ATP molecule, 8% more oxygen is needed during fatty acid oxidation than during glucose oxidation, one may argue that the muscles of young-adult mice on a HFD have a larger oxygen demand, something opposite to the pathological cardiac hypertrophy that is associated with a shift from fatty acid to glucose metabolism to overcome energy starvation (van Bilsen et al., 2004). It has therefore been suggested that to match oxygen supply with increased oxygen demand, a HFD promotes angiogenesis in addition to mitochondrial biogenesis, something reported by others (Silvennoinen et al., 2013) and in the present study, as reflected by the elevated C:F and LCFR.

The absence of HFD-induced angiogenesis (no significant rise in LCFR) in the EDL and diaphragm of the old mice with no change in oxidative capacity (reflected by similar SDH-OD) appears to support the notion that HFD-induced angiogenesis may serve to ensure an adequate oxygen supply in the young-adult mice on a HFD. However, this increased demand for oxygen cannot be the sole explanation, as the increased oxidative capacity in the EDL of young-adult mice was not accompanied by angiogenesis, and in the soleus of old mice, there was angiogenesis without a significant rise in oxidative capacity. Thus, these HFD-induced changes in oxidative capacity and capillarization appear to be uncoupled.

The uncoupling between changes in capillary supply and oxidative capacity of a fibre confirms our previous observation that the oxidative capacity does not determine the capillary supply to a fibre but rather fibre size (Bosutti et al., 2015). Our data support this relationship as in all cases where an increase in fibre size occurred, there was also angiogenesis, to such an extent that the overall capillary density and the capillary density per fibre (CFD) did not differ significantly between animals on a control diet and a HFD, except for a reduction in the diaphragm from young-adult mice. The significance of fibre size and the small contribution of oxidative capacity to the capillary supply to a fibre was also reflected by a stepwise regression that showed that 67% of the variation in the capillary supply to a fibre was explained by fibre size, with an additional 11% by muscle of origin, and only 0.7% by oxidative capacity (SDH-OD). Similar to what we have observed during ageing (Barnouin et al., 2017; Messa et al., 2019), the relationship between capillary supply with fibre size, muscle of origin and oxidative capacity was essentially unaltered by HFD (0.7% of the variation explained). This suggests that other functions of the microcirculation, such as removal of heat and waste products and substrate delivery, are more important than oxygen delivery.

Another factor that has not been considered in studies of HFD-induced obesity is the heterogeneity of capillary spacing, reflected by the logarithmic standard deviation of the capillary supply areas (log*_R_*SD) (Hoofd et al., 1985). Increased heterogeneity of the capillary spacing plays a role in the oxygenation of the tissue (Barnouin et al., 2017; Degens et al., 2006). The similar log*_R_*SD in the muscles shows that angiogenesis following a HFD does not occur at random but rather maintains the distribution of capillaries to preserve the potential for adequate intramuscular oxygenation.

To investigate the relationship between supply and demand further, we estimated the maximal oxygen demand of a fibre as the integrated SDH activity (FCSA×SDH-OD) and calculated the capillary supply (LCFR) to demand ratio (LCFR/SDH-Int) for each fibre. Following a HFD, the capillary supply to oxygen demand decreased in all muscles. This decreased ratio suggests a developing oxygen supply–demand mismatch during a HFD, similar to that seen in cardiac hypertrophy in rats (Des Tombe et al., 2002), suggesting the capillary supply is in deficit to oxygen capacity following a high-fat feeding. It is possible that after extended periods of HFD, capillary rarefaction occurs, something seen in muscles of obese people, which potentially could further aggravate the mismatch between oxygen supply and demand (Gomes et al., 2017).

### Conclusion

The data of the present study show that the muscles of old mice are more susceptible to HFD-induced changes in morphology than those in young-adult mice. The adaptations are muscle specific, with an increase in fibre size in the soleus but no change in the EDL. The increase in oxidative capacity is uncoupled from the HFD-induced angiogenesis but the angiogenesis is explicable by increases in fibre size. It therefore appears that many of the HFD-induced changes in muscle morphology, except the rise in IMCLs, are a consequence of additional loading of the muscle, rather than a direct effect of a HFD. It remains to be seen how longer durations of HFD and obesity affect muscle morphology, as caspase activation will over time result in muscle wasting (Kob et al., 2015) and muscle function already shows signs of impairment (Hill et al., 2019; Hurst et al., 2019).
